# Supplementary material for: Comparative genomics provides new insights into the diversity, physiology, and sexuality of the only industrially exploited tremellomycete: Phaffia rhodozyma
Source: BMC Genomics. 2016 Nov 9;17:901. doi: 10.1186/s12864-016-3244-7 (PMC5103461; doi:10.1186/s12864-016-3244-7)
Supplement: Additional file 6: — List of orphan genes with links to PFAM (related to Additional file 1: Table S1). (ZIP 1428 kb) [file 12864_2016_3244_MOESM6_ESM.zip › PFAM_UNIQUE/G01725_P.html]

#                                                                            --- full sequence --- -------------- this domain -------------   hmm coord   ali coord   env coord
  
# target name        accession   tlen query name           accession   qlen   E-value  score  bias   #  of  c-Evalue  i-Evalue  score  bias  from    to  from    to  from    to  acc description of target
  
#------------------- ---------- ----- -------------------- ---------- ----- --------- ------ ----- --- --- --------- --------- ------ ----- ----- ----- ----- ----- ----- ----- ---- ---------------------
  
G01725\_P             -            207 Borrelia\_orfA        PF02414.10   290       1.4    6.6   6.4   1   1   0.00094       1.9    6.2   6.4   131   169    55    91    29   113 0.49 - Borrelia ORF-A  
G01725\_P             -            207 bPH\_4                PF06713.6     74       0.3    9.7   4.7   1   2     0.033     2e+02    0.7   0.8    22    30    69    77    54    83 0.60 - Bacterial PH domain  
G01725\_P             -            207 bPH\_4                PF06713.6     74       0.3    9.7   4.7   2   2   2.8e-05      0.17   10.5   0.3    19    69    81   130    79   134 0.88 - Bacterial PH domain  
G01725\_P             -            207 DUF1319              PF07028.6    126     0.043   12.7   2.4   1   1     8e-05      0.08   11.8   2.4     9    82    22    94    16   102 0.69 - Protein of unknown function (DUF1319)  
G01725\_P             -            207 DUF2951              PF11166.3     98       1.5    7.4   6.8   1   1   0.00042       2.5    6.7   6.8    23    74    39    90    33    97 0.77 - Protein of unknown function (DUF2951)  
G01725\_P             -            207 Dynamin\_N            PF00350.18   168     0.014   13.8   0.3   1   1   0.00013     0.018   13.6   0.3    24    96    24    97    20   167 0.73 - Dynamin family  
G01725\_P             -            207 RICH                 PF05062.7     82       0.2   10.5   6.8   1   1   0.00012      0.35    9.7   6.8    26    71    49    90    33    97 0.74 - RICH domain  
G01725\_P             -            207 YukC                 PF10140.4    359     0.069   10.4   0.9   1   1   4.5e-05      0.09   10.0   0.9    40    90    60   110    41   125 0.81 - WXG100 protein secretion system (Wss), protein YukC
